# Either Rap1 or Cdc13 can protect telomeric single-stranded 3′ overhangs from degradation *in vitro*

**DOI:** 10.1038/s41598-019-55482-3

**Published:** 2019-12-16

**Authors:** Rikard Runnberg, Saishyam Narayanan, Humberto Itriago, Marita Cohn

**Affiliations:** Department of Biology, Genetics group, Lund University, Lund, Sweden

**Keywords:** Telomeres, DNA metabolism

## Abstract

Telomeres, the DNA-protein structures capping the ends of linear chromosomes, are important for regulating replicative senescence and maintaining genome stability. Telomeres consist of G-rich repetitive sequences that end in a G-rich single-stranded (ss) 3′ overhang, which is vital for telomere function. It is largely unknown how the 3′ overhang is protected against exonucleases. In budding yeast, double-stranded (ds) telomeric DNA is bound by Rap1, while ssDNA is bound by Cdc13. Here, we developed an *in vitro* DNA 3′end protection assay to gain mechanistic insight into how *Naumovozyma castellii* Cdc13 and Rap1 may protect against 3′ exonucleolytic degradation by Exonuclease T. Our results show that Cdc13 protects the 3′ overhang at least 5 nucleotides (nt) beyond its binding site, when bound directly adjacent to the ds-ss junction. Rap1 protects 1–2 nt of the 3′ overhang when bound to dsDNA adjacent to the ds-ss junction. Remarkably, when Rap1 is bound across the ds-ss junction, the protection of the 3′ overhang is extended to 6 nt. This shows that binding by either Cdc13 or Rap1 can protect telomeric overhangs from 3′ exonucleolytic degradation, and suggests a new important role for Rap1 in protecting short overhangs under circumstances when Cdc13 cannot bind the telomere.

## Introduction

Telomeres are specialized nucleoprotein structures localized at the ends of linear chromosomes that protect and stabilize the genome. Telomeric DNA consists of repetitive G-rich sequences that extend in the 5′ to 3′ direction as double-stranded (ds) DNA and beyond into a single-stranded (ss) 3′ overhang^1,2^. Highly specific telomere binding proteins interact with both the dsDNA and ssDNA in order to protect them from harmful DNA damage responses^3–6^. Due to the limitations of the DNA replication machinery, telomeres shorten with each replication cycle^7^. Most eukaryotes prevent telomere shortening, which otherwise leads to cellular senescence^8^, through extension of the 3′ overhang by telomerase reverse transcriptase^9–11^. In humans, telomerase maintains telomeres in germ line and stem cells, while most somatic cells do not express telomerase^12^. Re-activation of telomere lengthening, as well as genomic instability at unprotected telomeres, contributes to the development of cancer^13^.

The telomeric 3′ overhang is important as the substrate for telomerase and as the binding site for protective proteins^3,6,14^. Furthermore, in human cell lines without telomerase activity, telomere 3′ overhang length is proportional to the rate of telomere shortening per cell division^15^. Hence, the regulation of telomere 3′ overhang length might have implications for cell senescence and aging. Telomeric 3′ overhangs are also required for the formation of higher-order DNA structures that promote telomere protection, such as G-quadruplexes and telomere loops (t-loops)^16,17^. In higher eukaryotes, t-loop formation involves the fold back and invasion of the 3′ overhang into the telomeric double-stranded region of the same chromosomal end, providing protection by effectively sequestering the 3′ overhang^18^. The length of telomeric 3′ overhangs varies between species. The budding yeast *Saccharomyces cerevisiae* has short overhangs of 12–14 nucleotides (nt) for most of the cell cycle, with longer (>30 nt) overhangs forming during S-phase^2,19^. The length of human telomeric 3′ overhangs ranges from 12 up to several hundred nt^20–22^ and at least 6 nt was shown to be required for the formation of a t-loop structure *in vitro*^23^.

In order to generate 3′ overhangs, telomeres are subject to 5′ end resection, C-strand fill in, and extension by telomerase^24–26^. Previous studies have focused on identifying and understanding the regulation of exonucleases acting on the 5′ end, which are needed to create the 3′ overhang. However, the 3′ overhang is also at the risk of being degraded by 3′ exonucleases, and 3′ trimming has been suggested as a means to generate preferred 3′ end permutations in some species^27,28^. A large number of 3′ exonucleases are present in eukaryotic cells^29^, yet few studies have specifically addressed how they might act at the telomere 3′ overhang.

We have developed an *in vitro* assay to study the protection of *Naumovozyma castellii* telomeres from 3′ exonucleolytic degradation, using *E. coli* Exonuclease T/RNase T (ExoT) as a model exonuclease. ExoT belongs to the DEDD family of 3′ exonucleases^29^. Members of this family of 3′ exonucleases include the exonuclease activities of DNA polymerases, the WRN helicase which has a function in telomeric DNA processing^30,31^, and the major mammalian 3′ exonucleases TREX1 and TREX2^29,32,33^. Intriguingly, WRN helicase activity is necessary for resolution of t-loops and may have a role in preventing 3′ overhang shortening *in vivo*^31^. Hence, the commercially available 3′ exonuclease ExoT is suitable for an *in vitro* model of telomere 3′ end protection.

*N. castellii* is a budding yeast of the Saccharomycetaceae family. Due to its regular short repetitive telomeric repeats (5′-TCTGGGTG-3′) it facilitates precise studies of telomere binding proteins^34^. *N. castellii* telomeric 3′ overhangs are highly variable in length, ranging from the minimal measurable size of 14 nt up to 200 nt in unsynchronized cells^35^. Hence, *N. castellii* telomeric repeats and 3′ overhangs resemble those of humans. *N. castellii* homologs of the budding yeast telomeric ssDNA binding protein Cdc13^36^, and telomeric dsDNA binding protein Rap1^37^, have previously been characterized.

Cdc13 recruits telomerase to the telomere end in *S. cerevisiae*^6^, and is part of the three membered complex CST, which in both humans and budding yeast functions in regulating telomere overhang length^38,39^. *N. castellii* Cdc13 binds telomeric ssDNA with high affinity through an 8 nt minimal binding site (MBS) 5′-GTGTCTGG-3′^36^.

Rap1 is conserved as a telomere associated protein across multiple species, including vertebrate animals, interacting with human telomeres via telomere binding protein TRF2^40,41^. *S. cerevisiae* Rap1 binds telomeric dsDNA directly^42^ and is an essential protein required for different telomere functions, including silencing^43^, telomere length regulation^44^, protection against telomere-telomere fusion^45^ and preventing excessive 5′ end resection^46^. *N. castellii* Rap1 binds to the 12 bp MBS 5′-GGGTGTCTGGGT-3′ on telomeric dsDNA^37^. Binding occurs via two Myb-subdomains, and the MBS is divided in two half-sites (5′-GGTGT-3′ and 5′-TGGGT-3′) separated by one base redundant for high affinity binding^47^. *N. castellii* Rap1 has been found to also bind across the ds-ss junction of certain 5′ end permutations, causing the Rap1 and Cdc13 binding sites to partly overlap on some ds-ss junctions^48^.

Previously we have used an *in vitro* assay to evaluate how Rap1 and Cdc13 protect telomeres from 5′ exonucleolytic degradation, showing that both proteins can protect the 5′ end even when bound at some distance from the ds-ss junction^49^. In the work presented here, we have evaluated the ability of Cdc13 and Rap1 proteins to protect the telomeric 3′ overhang from 3′ exonucleolytic degradation through the use of an *in vitro* assay that utilizes recombinant *N. castellii* Cdc13 and Rap1, synthetic telomere mimicking DNA substrates and *E. coli* Exonuclease T. The homogenous telomeric repeats of *N. castellii* enable us to study the protection by the proteins at their exact binding sites relative to the ds-ss junction and the 3′ end.

## Results

### Cdc13 protects the telomeric 3′ overhang against 3′ exonucleolytic degradation

**Figure 1:**
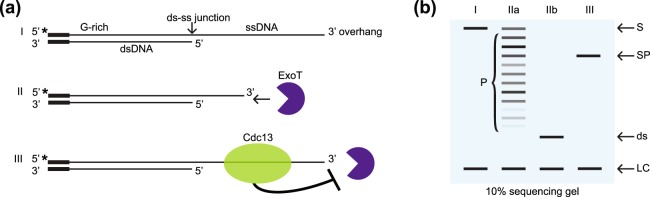
Schematic illustration of the DNA End Protection Assay (DEPA) on the 3′ overhang. (**a**) DNA oligonucleotides are annealed to form model telomeres with a double-stranded part and a single-stranded 3′ overhang (I). All oligonucleotides contain a short non-telomeric guide sequence to ensure efficient annealing while the telomeric part is varied to create different length overhangs and different 5′ end permutations. Exonuclease T (ExoT) removes nucleotides in the 3′ to 5′ direction of the single-stranded 3′ overhang (II) of the G-rich strand which is radioactively 5′ end labelled (*). To assay for 3′ end protection, Cdc13 is pre-bound to the telomere before adding ExoT to the reaction, which will inhibit the exonuclease (III). (**b**) Reactions are stopped at different incubation times, de-proteinized, a labelled oligonucleotide loading control (LC) is added, samples are ethanol precipitated and run on a denaturing 10% polyacrylamide sequencing gel. Initially, the uncleaved substrate (S) is visible at the top of the gel (I) but as the exonuclease reaction progresses, products of decreasing size (P) appear on the gel while the substrate diminishes (IIa). Lane I, no enzyme control showing the initial substrate (S) and the loading control (LC); lane IIa, possible digestion products at different incubation times; lane IIb, ds product after the substrate 3′ overhang has been fully digested; lane III, a reaction where the substrate was pre-incubated with either Cdc13 or Rap1, providing substrate protection (SP) which generates a product of a certain length due to enzyme stalling.

In this study we sought to assess the ability of the telomere binding proteins Cdc13 and Rap1 to protect the single-stranded 3′ overhang from 3′ exonucleolytic degradation using the commercially available Exonuclease T (ExoT) in a technique we have named the 3′ DNA End Protection Assay (3′DEPA). There are several enzymes with 3′ exonucleolytic activity *in vivo*, many of which participate in important events regarding the replication, proof-reading and repair of DNA. For this assay, ExoT was selected as a commercially available enzyme that removes nucleotides in the 3′ to 5′ direction, generating blunt ends in DNA molecules with a 3′ ssDNA extension^50^. The budding yeast *N. castellii* has an 8 nt regular telomere repeat which allows us to study the positional effect of the telomere binding proteins around the ds-ss junction of the telomere. The oligonucleotides used in the 3′DEPA contain the *N. castellii* telomeric repeat sequence and a short non-telomeric guide sequence. The annealing of the oligonucleotides produces a substrate that has a double-stranded region and a protruding 3′ overhang, mimicking the *N. castellii* telomere (I in Fig. 1a). The 5′ end of the G-rich strand of the substrate is radioactively labelled for its visualization on a sequencing gel. ExoT will gradually degrade the single-stranded 3′ overhang until it reaches the double-stranded region, generating a blunt end (II in Fig. 1a). To assess the protection by the telomere binding proteins, the substrate is pre-incubated with the protein, i.e. Cdc13, before ExoT is added (III in Fig. 1a). Samples of the reaction are stopped at different incubation times and resolved together on a sequencing gel (I-III in Fig. 1b). The protection provided by the telomere binding proteins is evaluated based on the length of the uncleaved 3′ overhang in comparison to the fully digested 3′ overhang of a BSA (non-DNA binding) protein control.

**Figure 2:**
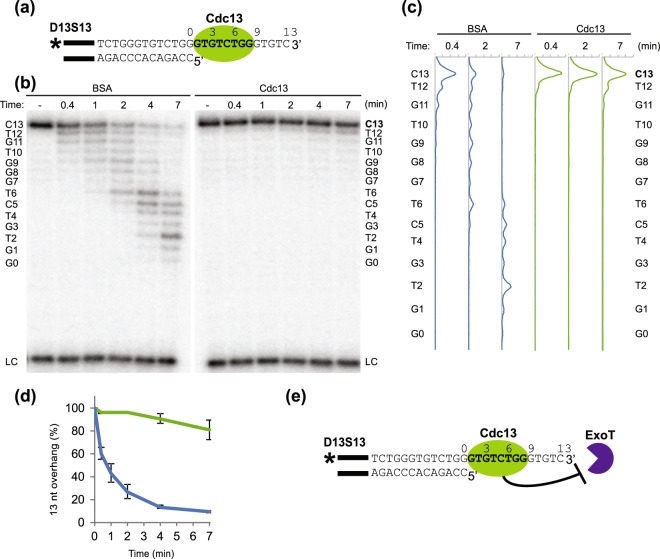
DNA 3′ end protection when Cdc13 is bound immediately adjacent to the ds-ss junction. (**a**) Schematic figure indicating the interaction of Cdc13 with telomeric substrate D13S13. Black bars indicate a 14 nt guide sequence used to ensure proper annealing of the two strands, bold text indicates Cdc13 MBS, “*” indicates ^32^P radioactive label used for detection. Numbers above the sequence denotes the distance in nt relative the ds-ss junction. (**b**) Sequencing gel showing 3′DEPA products of D13S13 incubated with 0.015 U/μl ExoT for 25 s, 1 min, 2 min, 4 min and 7 min, following pre-incubation with BSA or Cdc13 as indicated. “–” indicates no enzyme added, “LC” loading control. The sequence of the overhang is written beside the gel and numbered from the ds-ss junction (G0) to the 3′ end (C13). (**c**) Lane profiles showing the intensities of each band in the lanes, corresponding to the indicated time points for each graph, after pre-incubation with Cdc13 (green line) or the BSA control (blue line). The sequence corresponding to each peak is written as in (**b**). (**d**) The proportion of overhangs at 13 nt (full length) was quantified at each reaction time for Cdc13 containing reactions (green line) and BSA control (blue line). Error bars indicate standard error of mean (SEM) for n = 3 experiments. (**e**) Schematic figure showing the protection from degradation by ExoT which was exerted by the binding of Cdc13 to the 3′ overhang.

Cdc13 is the major telomere ssDNA binding protein in budding yeast, and binding of Cdc13 to the telomeric 3′ overhang is essential for the overall maintenance and protection of the telomere^39^. When Cdc13 binds the 3′ overhang it is expected to provide protection against 3′exonucleases by steric hindrance. However, the role of Cdc13 in protecting the telomeric overhang from 3′ exonucleases has not yet been characterized at the molecular level *in vitro*. We set out to do so by employing the 3′DEPA with a telomeric probe containing 13 nt of dsDNA and 13 nt of ssDNA *N. castellii* telomeric sequence, named D13S13 (where the nomenclature DxSy is used throughout this work to denote that there are x number of base pairs of telomeric dsDNA and y number of ssDNA nucleotides in the telomeric 3′ overhang). This substrate contains a Cdc13 minimal binding site (MBS) immediately adjacent to the ds-ss junction (Fig. 2a). The substrate was either pre-incubated with Cdc13 protein or with BSA protein as a negative control, before the addition of the ExoT. Electrophoretic Mobility Shift Assay (EMSA) confirmed the binding of Cdc13 to D13S13 under the conditions used for 3′DEPAs (Supplementary Fig. S1). As expected, when D13S13 was pre-incubated with BSA, ExoT digested the 3′ overhang progressively up to the ds-ss junction, visible on the sequencing gel as a ladder of shorter and shorter reaction products until reaching the length of the ds part of D13S13 (left part of Fig. 2b,c). In contrast, when D13S13 was pre-incubated with Cdc13, the full length 3′ overhang (13 nt) remains almost completely unaltered throughout the reaction, despite the presence of the ExoT (right part of Fig. 2b,c). Few products below the 13 nt overhang accumulate at the longest incubation time (last lane/profile Fig. 2b,c), suggesting that binding of Cdc13 strongly hinders the digestion of ssDNA by ExoT. Quantification of the percentage of full length substrate that remains uncleaved after increasing incubation times shows that Cdc13 binding results in nearly complete protection of the 3′ overhang, while in the BSA control the overhang is almost completely degraded by ExoT (Fig. 2d). Thus, Cdc13 provides protection of at least 5 nt beyond its MBS when bound immediately adjacent to the ds-ss junction.

To confirm the protection provided by Cdc13 is due to its binding of D13S13, rather than an unspecific inhibition of ExoT activity, control experiments were performed where an additional ssDNA non-telomeric oligonucleotide without a Cdc13 binding site was added to the reactions (Supplementary Fig. S2). This showed that while D13S13 is well protected by Cdc13, an unbound oligonucleotide is not. Hence, the protection is dependent on Cdc13 binding the substrate.

In summary, from these experiments it can be concluded that *N. castellii* Cdc13 bound to the telomeric 3′ overhang can protect it from 3′ exonucleolytic degradation by ExoT, allowing for the preservation of 3′ overhangs at least 13 nt long (Fig. 2e).

### Rap1 protects 1–2 nt of the 3′ overhang when binding to dsDNA adjacent to the ds-ss junction

**Figure 3:**
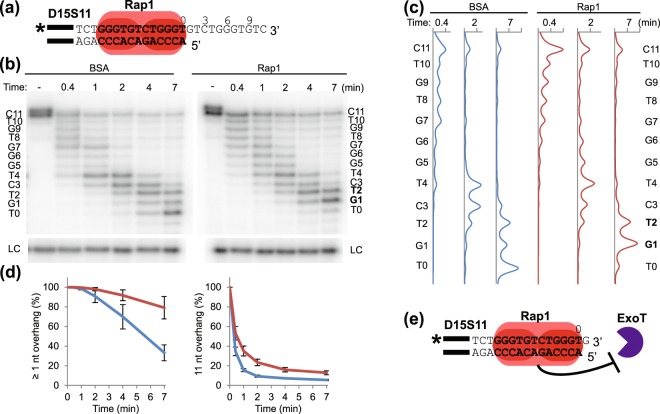
DNA 3′ end protection when Rap1 is bound immediately adjacent to the ds-ss junction. (**a**) Schematic figure indicating the interaction of Rap1 with telomeric substrate D15S11. Black bars indicate the 14 nt annealing guide sequence, bold text indicates Rap1 MBS, “*” indicates ^32^P radioactive label. Numbers above the sequence denotes the distance in nt relative the ds-ss junction. (**b**) Sequencing gel showing 3′DEPA products of D15S11 incubated with 0.015 U/μl ExoT for 25 s, 1 min, 2 min, 4 min and 7 min, following pre-incubation with BSA or Rap1 as indicated.“–” indicates no enzyme added, “LC” loading control. The sequence of the 3′ overhang is written beside the gel and numbered from the ds-ss junction (T0) to the 3′ end (C11). The G1 and T2 bases protected by Rap1 are in bold. (**c**) Lane profiles showing the intensities of each band in the lanes, corresponding to the indicated time points for each graph, after pre-incubation with Rap1 (red line) or the BSA control (blue line). The sequence corresponding to each peak is written as in (**b**). (**d**) The proportion of overhangs at lengths of ≥1 nt and 11 nt quantified at each reaction time for Rap1 containing reactions (red line) and BSA control (blue line). Error bars indicate SEM for n ≥ 5 experiments. (**e**) Schematic figure showing the 1 nt 3′ overhang that Rap1 protects from degradation by ExoT when bound adjacent to the ds-ss junction. Uncropped gel is shown in Supplementary Fig. S3.

We recently showed that Rap1 can protect telomeric 5′ ends from exonucleolytic degradation, even when bound several base pairs inwards of the ds-ss junction, indicating that Rap1 provides enough steric hindrance to protect the telomere from exonucleases even at some distance^49^. Thus, we hypothesised that Rap1 may also protect part of the 3′ overhang form exonucleases when bound to telomeric dsDNA. In order to test this hypothesis, 3′DEPA using the telomeric substrate D15S11 was performed (Fig. 3a, uncropped gel in Supplementary Fig. S3). This substrate contains 15 bp of telomeric dsDNA and an 11 nt telomeric ssDNA 3′ overhang, and encompasses a Rap1 MBS immediately adjacent to the ds-ss junction (Fig. 3a). As expected, ExoT gradually digested the entire 3′ overhang of D15S11 in control reactions containing non-DNA binding BSA protein (left part of Fig. 3b,c). When D15S11 instead was pre-bound by Rap1, the reaction rate was slightly slower than the control reaction, and the major end-products corresponded to 1–2 nt long ssDNA overhangs (right part of Fig. 3b,c). The relative amounts of different overhang lengths remaining at each time point in these experiments was quantified, showing that the reaction rate of Rap1 containing reactions is only marginally lower than that of BSA, and at distal parts of the overhang little protection is seen (Fig. 3d and Supplementary Fig. S4). However, at 1–2 nt positions of the overhangs (1–2 nt from the Rap1 MBS) considerably more protection is seen (red versus blue lines in the left graph of Fig. 3d). This indicates that at least the innermost nucleotide of the 3′ overhang, 1 nt beyond the Rap1 MBS, is being protected by Rap1 (Fig. 3e).

Hence, Rap1 is able to protect 1–2 nt of telomeric ssDNA outside of its MBS when positioned right next to the ds-ss junction. This suggests that Rap1 does not provide a major steric hindrance to 3′ exonucleases. Nevertheless, the slight slowdown of the reaction rate and the marked protection of the innermost 1–2 nt of the overhang might still be physiologically relevant.

### Rap1 confers a more extensive protection of the 3′ overhang when bound to an alternative binding site across the ds-ss junction

The canonical MBS of Rap1 consists entirely of dsDNA, however an alternative Rap1 binding site across the ds-ss junction was recently discovered by our group^47,48^. In this binding mode Rap1 recognizes the same dsDNA sequence as in the canonical binding mode, except part of the dsDNA is replaced with the ssDNA of the 3′ overhang^48^. The biological role of this alternative binding mode is still unclear. Since Rap1 interacts with ssDNA of the telomeric 3′ overhang when bound across the ds-ss junction, we hypothesised that Rap1 might be able to better protect the 3′ overhang from exonucleolytic degradation when utilizing this binding mode, compared to when bound only to dsDNA.

**Figure 4:**
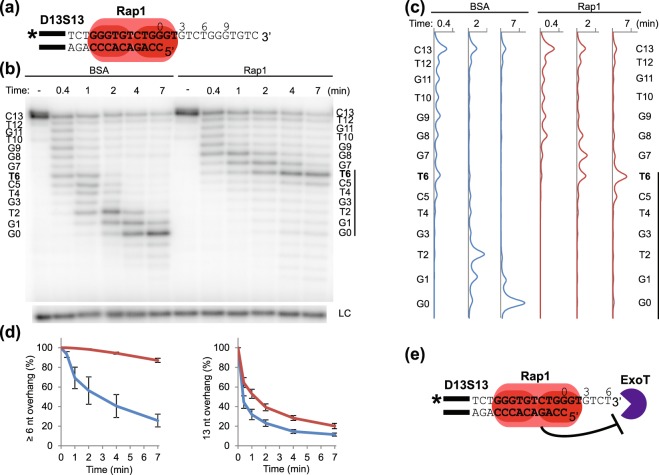
DNA 3′ end protection when Rap1 is bound across the ds-ss junction. (**a**) Schematic showing the interaction of Rap1 with telomeric substrate D13S13. Black bars indicate the 14 nt annealing guide sequence, bold text indicates Rap1 MBS, “*” indicates ^32^P radioactive label. Numbers denote the distance in nt relative the ds-ss junction. (**b**) Sequencing gel showing 3′DEPA products of D13S13 incubated with 0.015 U/μl ExoT for 25 s, 1 min, 2 min, 4 min and 7 min, following pre-incubation with BSA or Rap1 as indicated.”–“ indicates no enzyme added, “LC” loading control. The sequence of the 3′ overhang is written beside the gel and numbered from the ds-ss junction (G0) to the 3′ end (C13). The stalling point at T6 is shown in bold and the bar indicates the area of the overhang protected by Rap1. (**c**) Lane profiles showing the intensities of each band in the lanes, corresponding to the indicated time points for each graph, after pre-incubation with Rap1 (red line) or the BSA control (blue line). The sequence corresponding to each peak is written as in (**b**). (**d**) The proportion of overhangs at lengths of ≥6 nt and 13 nt was quantified at each reaction time for BSA (blue line) and Rap1 (red line) containing reactions. Error bars indicate SEM for n ≥ 6 experiments. (**e**) Schematic figure showing the 6 nt 3′ overhang that Rap1 protects from degradation by ExoT when bound across the ds-ss junction. Uncropped gel is shown in Supplementary Fig. S3.

In order to test this hypothesis, 3′DEPAs were performed using telomeric substrate D13S13, consisting of 13 bp dsDNA and a 13 nt ssDNA 3′ overhang (Fig. 4a). The 5′ end permutation of D13S13 allows Rap1 binding across the ds-ss junction although 2 nt of its binding site sequence reach into the ssDNA (Fig. 4a, uncropped gel in Supplementary Fig. S3). As previously, when D13S13 is pre-incubated with BSA, 3′exonucleolytic degradation by ExoT gradually progresses until the entire 3′ overhang is completely digested (left part of Fig. 4b,c). However, when D13S13 is pre-bound with Rap1 the reaction slows down, initially at 8 nt from the ds-ss junction, then at later time points products accumulate mainly at 6 nt from the ds-ss junction, i.e. 4 nt distal of the Rap1 MBS sequence (right part of Fig. 4b,c). Quantification of the relative amounts of reaction products corresponding to various overhang lengths was performed (Fig. 4d and Supplementary Fig. S5). This showed that indeed a majority of the ExoT digestion products are ≥6 nt long (+4 nt from Rap1 MBS) when D13S13 is bound by Rap1, while in the BSA control reactions, most of the 3′ overhangs were degraded to a shorter length than 6 nt at the same time points (Fig. 4d, left graph, difference between blue and red lines). At the most distal part of the 3′ overhang, little difference is seen between BSA and Rap1 containing reactions (Fig. 4d, right graph). This indicates that the hindrance of ExoT by Rap1 is mainly confined to an area 6 nt distally of the ds-ss junction, i.e. +4 nt from the Rap1 MBS (Fig. 4e). Notably, when using higher concentrations of ExoT and longer incubation times, the protection of 6 nt is seen at all time points tested (Supplementary Fig. S6). Although the hindrance by Rap1 is eventually partly overcome, it is only at time points far beyond those where the overhangs in the BSA control reactions are fully digested (Supplementary Fig. S6).

To ensure that the observed hindrance of ExoT by Rap1 is indeed mediated through Rap1 interaction with DNA, control 3′DEPA experiments were performed using a similar substrate having a mutated Rap1 binding site; D13S13mutRap1 (Supplementary Fig. S7). EMSA showed that only minute amounts of Rap1 could bind this substrate (Supplementary Fig. S7). Only a very slight slowdown of the reaction rate could be observed when D13S13mutRap1 was pre-incubated with Rap1 compared to BSA, and pre-incubation with Rap1 did not produce any product pattern indicative of enzyme stalling. This shows that Rap1 indeed needs to bind the substrate with high affinity in order to protect the overhang from ExoT degradation. Further highlighting the importance of DNA binding, 3′DEPAs performed with the isolated Rap1 DNA-binding domain (DBD) gave a similar degradation pattern as full length Rap1 when bound to D15S11 or D13S13 (Supplementary Fig. S8). This indicates that protection against 3′ exonucleolytic degradation is primarily mediated by the Rap1 DBD.

Taken together, our results show that indeed Rap1 is able to provide protection of telomeric ssDNA 3′ overhangs. However, when bound at different specific locations in relation to the ds-ss junction, Rap1 contributes vastly differently to the protection. Strikingly, Rap1 confers a significant protection of the 3′ overhang when bound with high affinity across the ds-ss junction. In this binding mode, where the MBS sequence reaches 2 nt into the single-stranded 3′ overhang, Rap1 protects at least 6 nt of ssDNA. The difference in protection ability may indicate that Rap1 is forming different interactions with the DNA when bound across the ds-ss junction.

## Discussion

We have studied the role of *N. castellii* Cdc13 and Rap1 in the protection of telomeric 3′ overhangs against 3′ exonucleolytic degradation. We found that Cdc13 efficiently stops degradation of the 3′ overhang by *E. coli* ExoT, protecting at least 5 nt distal of the Cdc13 minimal binding site (MBS) (Fig. 2). Interestingly, our assays show that Rap1 is also able to protect the 3′ overhang. When bound immediately adjacent to the ds-ss junction, Rap1 prevents ExoT from degrading the innermost 1–2 nt of the 3′ overhang (Fig. 3). Protection by Rap1 is extended considerably when it is bound across the ds-ss junction. At a permutation where two bp of dsDNA in the Rap1 MBS are replaced for ssDNA, protection extends to encompass a 6 nt overhang (Fig. 4).

Few studies prior to this one have specifically addressed how telomere binding proteins prevent telomere overhang degradation by 3′ exonucleases, and to our knowledge this is the first *in vitro* assay addressing this aspect of telomere end processing in budding yeast. Since it is not clear which endogenous 3′ exonucleases might act at budding yeast telomeres *in vivo*, we used the commercially available *E. coli* ExoT as a model 3′ exonuclease. While this model may not take some aspects (such as protein-protein interactions and sequence bias) of a hypothetical telomere specific endogenous 3′ exonuclease into account, it does clearly show that both Cdc13 and Rap1 have the potential to protect the telomere 3′ end from exonucleolytic degradation. Furthermore, the methodology can be applied to study the ability of other telomeric proteins to protect the 3′ overhang, like the Rap1-interacting proteins Rif1 and Rif2^51^.

Cdc13 is the core telomere ssDNA binding protein in budding yeast, making it an obvious candidate for blocking 3′ exonucleases at the telomere overhang. Our results show that indeed, binding by Cdc13 is enough to stop the action of a 3′ exonuclease. This allows us to speculate about the situation *in vivo*; Cdc13 should be able to protect most conceivable 3′ overhangs if they are long enough to contain a Cdc13 binding site, and if the overhang is long enough to contain multiple binding sites, the most distally positioned Cdc13 is expected to prevent 3′ exonucleases from degrading the overhang. A similar study was previously performed for humans, where the telomere ssDNA binding protein POT1 was shown to prevent degradation by the Werner syndrome helicase WRN, that contains a 3′ exonuclease activity that can specifically degrade human telomere overhangs^52^. Hence, *N. castellii* Cdc13 may have a similar role in preventing telomeric 3′ end degradation as POT1 does in humans.

Cdc13 must bind the overhang to protect it. One situation where it cannot bind is when the overhang is too short to encompass a Cdc13 MBS, such as D15S11 (Fig. 3). Cdc13 binding may also be prevented if it is out-competed by Rap1. This can occur on a short overhang that only contains one Cdc13 MBS positioned at a 5′ end permutation where Rap1 can bind across the ds-ss junction and compete for binding, such as D13S13 (Fig. 4)^48^. Our results suggest that in both these situations Rap1 can provide protection of the 3′ overhang. Even though the overhangs protected by Rap1 are shorter than those protected by Cdc13, it may still have physiological relevance. It was recently shown that a 6 nt primer, similar to the overhang protected by Rap1 on the D13S13 substrate studied here, can be extended by *N. castellii* telomerase^53^. Even the short 1–2 nt overhang protected by Rap1 when bound only to dsDNA (D15S11, Fig. 3), might be enough to aid the generation of a longer overhang, by improving the efficiency of 5′ end resection. Since Rap1 binding would prevent access by telomerase to the overhang, or access by 5′ exonucleases to the 5′ end^49^, removal of Rap1 might be necessary for further processing of the overhang. The 3′ overhang protection by Rap1 shown in this study, might thus reflect a temporary state in telomere end processing, where Rap1 prevents catastrophic loss of DNA before the telomere ends are further processed by other proteins.

Notably, the extended 3′ end protection by Rap1 when bound across the ds-ss junction, as compared to when bound only to dsDNA, does not only correspond to the additional 2 nt at the proximal part of the 3′ overhang. Rather, it is an increased protection relative to the Rap1 MBS, so that it protects 4 nt distal of the MBS instead of 1–2 nt. This suggests that the conformation of the protein might be altered when bound to the ds-ss junction, providing additional steric hindrance and/or additional interactions with more distal parts of the overhang. The fact that the Rap1 DBD on its own can provide 3′ end protection suggests that neither additional DNA interactions nor steric hindrance provided by other parts of the protein are required for inhibiting 3′ exonucleolytic degradation by ExoT (Supplementary Fig. 8).

3′DEPA experiments with Rap1 DBD were also performed using DBD variants that had C-terminal truncations of the wrapping loop and latch regions (Supplementary Fig. S8). These regions help lock the two Rap1 Myb subdomains in place on dsDNA^54,55^, and our group recently showed that the ability of the Rap1 DBD to protect telomeric 5′ ends from exonucleolytic degradation is severely compromised when it lacks the wrapping loop and latch^49^. In contrast to this, we found that deletion of these regions does not affect the protection of the 3′ end (Supplementary Fig. S8). Hence, our results suggest that Rap1 does not need to be firmly locked in place on DNA to make the interactions with the 3′ overhang required for 3′ end protection. Instead, it may be that when bound across the ds-ss junction one of the Rap1 Myb subdomains has a looser attachment than when bound to dsDNA, enabling it to make additional interactions with ssDNA on more distal parts of the overhang than when it is fully bound to dsDNA. Intriguingly, such conformational changes to Rap1 when bound across the ds-ss junction could potentially be a mechanism for Rap1 to gain a separate function at the telomere end as compared to when bound to dsDNA further inward on the telomere.

The human Rap1 protein (hRap1) has also been shown to bind ds-ss junctions *in vitro*, in the absence of TRF2^56^. In complex with TRF2, hRap1 increases the selectivity of TRF2 to telomeric DNA and promotes its localization to telomeric ds-ss junctions, where it aids the formation of t-loops^23,57^. Although t-loop formation has not been reported for any wild-type budding yeast, we cannot exclude the possibility of their formation. Intriguingly, t-loop formation was observed in a mutant *Kluyveromyces lactis* strain modified to have telomeric repeats that are poorly bound by Rap1^58^. These uncapped elongated telomeres displayed long 3′ overhangs and increased *RAD52*-dependent recombination rates, which could suggest that Rap1 may play a role in repressing strand invasion by the 3′ overhangs. Thus, Rap1 may restrict the overhang access to the internal duplex region, thereby limiting the extent of different telomere-associated recombination activities, while at the same time protecting it from degradation. Since *N. castellii* has been shown to display long 3′ overhangs, it would be highly interesting to investigate whether t-loops may form in this species. Moreover, we recently showed that alternative lengthening of telomeres (ALT) in *N. castellii* telomerase-negative cells occur by a copying mechanism that is initiated at an interstitial telomeric sequence imbedded within the subtelomeric region^59^. Possibly, a t-loop structure could be involved in the mechanistics of the ALT process, thus allowing for the excision of t-circles that could act as templates for the telomere elongation.

In conclusion, we designed an *in vitro* exonuclease assay to assess the ability of *N. castellii* Rap1 and Cdc13 to protect the telomeric ss-overhang from 3′ exonucleolytic degradation and found that either one can protect the 3′ overhang. The redundancy of having two core telomere binding proteins with this function, suggests it is an important aspect of telomere protection and maintenance. Having telomeric 3′ overhangs of the right length is crucial for proper telomere function, and the mechanisms for generating and regulating telomeric 3′ overhangs has been extensively studied^2,20,25,26,39,46^, yet few studies have specifically addressed how 3′ exonucleases are prevented from degrading the overhang. Our results indicate that two of the core proteins involved in telomere protection and maintenance, Cdc13 and Rap1, may have complementary roles in preventing exonucleolytic degradation of the telomeric 3′ overhangs.

## Materials and Methods

### Oligonucleotides

Oligonucleotides were synthesised by Eurofins Genomics, either gel purified in house or by the manufacturer. Sequences of oligonucleotides used in this paper are listed below (Table 1).

**Table 1:** Oligonucleotides used in this study.

G-rich strand sequence (5′-3′)	used for preparing
*GTCACACGTCACAC*-TCTGGGTGTCTGGGTGTCTGGGTGTC	D13S13, D15S11
*GTCACACGTCACAC-*TCTGGGacTCTccGTGTCTGGGTGTC	D13S13mutRap1
**C-rich strand sequence (**5′**-**3′**)**	**used for preparing**
CCAGACACCCAGA-*GTGTGACGTGTGAC*	D13S13
ggAGAgtCCCAGA-*GTGTGACGTGTGAC*	D13S13mutRap1
ACCCAGACACCCAGA-*GTGTGACGTGTGAC*	D15S11
**Cdc13 no binding control sequence (**5′**-**3′**)**	**used for preparing**
GGACTTAAAATGGCGTGGCAGAACTAACTCTT	Control Supplementary Fig. S2

The respective G-rich and C-rich strands were annealed to generate the indicated telomeric substrates. Dx, Sy, in the substrate name indicates the number of telomeric double- and single-stranded nucleotides, respectively. Italic font indicates a 14 nt guide sequence used for directing the correct annealing. Lower case letters indicate alterations to telomeric sequence.

### Protein expression and purification

Expression and purification of *N. castellii* Cdc13, full length Rap1 and Rap1 DBD variants was performed as previously described^49^, with the addition that proteins were concentrated and buffer exchanged on Amicon Ultra centrifugal filters (Millipore) cut-off 10 kDA (Rap1) and 30 kDa (Cdc13).

### Electrophoretic mobility shift assay (EMSA)

EMSA was performed to assess protein binding and annealing of the substrates. EMSA was performed as previously described^48^, except the G-rich strand was radioactively labelled, annealing was performed with a 1.5 times excess of the C-rich (unlabelled) probe and all binding reactions contained 10 fmol of probe together with purified recombinant *N. castellii* Cdc13 or Rap1.

### 3′ DNA end protection assay (3′DEPA)

First, Cdc13 or Rap1 was allowed to bind the overhang substrate. BSA (diluted in the storage buffer of the respective proteins) was used as a non-DNA binding control in an identical reaction. The binding was performed under the same conditions used for EMSAs, except the reaction was scaled up to contain 97.5 μl, corresponding to 6.5 EMSA reaction volumes^48^. The concentrations used of Cdc13 and Rap1 were determined by EMSA to give ~60–100% shifted D13S13 or D15S11 probe (typically 0.9–3 μg Cdc13, 1.4–3.5 μg of full length Rap1, or 0.4–0.7 μg of Rap1 DBDs per EMSA reaction volume, depending on batch and fraction), while producing negligible amounts of higher than 1:1 stoichiometry complexes. Following binding, a 15 μl volume was removed from the reaction batch to be used as a “no enzyme added” control. Exonuclease T (ExoT, New England Biolabs #M0265S) was added to the remaining reaction batch (0.015 U/μl final concentration), which was then incubated at 25 °C. 15 μl volumes were removed from the reaction batch at the time points indicated in figure legends, mixed with 9.5 μl stop buffer (53 mM Tris-HCl pH 8.0, 53 mM EDTA pH 8.0, 1.3% SDS, 5.2 μg/μl Proteinase K) and incubated at 65 °C for at least 20 min. DNA products were then precipitated and separated by denaturing PAGE as previously described^49^, with the exception that a 19 nt radioactively labelled oligonucleotide was used as loading control.

### Image acquisition and quantification

Dried gels were exposed to phosphorimager screens and scanned using Typhoon FLA9500 imager (GE Life Sciences). Quantification was performed in the ImageQuant software (GE Life Sciences) from scans at 200 μm resolution (saved as GEL files). Lanes were manually selected, background removed using “rolling ball” with a radius of 200, bands automatically detected (minimum slope 10, median filter 4, % max peak 0), followed by manual inspection and correction if needed. For 3′DEPAs the data was compiled in a table containing the volume of each band in every lane, which was exported to Excel (Microsoft) where calculations and graphs were made. The product remaining at each time point was calculated by dividing the sum of band volumes corresponding to certain overhang lengths (e.g. the upper 8 bands for a ≥6 nt overhang on D13S13) with the sum of all the product band volumes. The value of the “no enzyme added” control was set to 100%. For EMSAs, the band% of the major shifts and the unbound probe were determined in ImageQuant, and % of bound probe calculated as total (100%) minus band% of unbound probe.

Lane profiles were generated in ImageQuant from GEL images scanned at 50 μm resolution by manually selecting lanes, which were exported as numerical values to Excel. Values were normalized to the loading control band volume of each lane, and values corresponding to the bands from the full length substrate through to the blunt ended product were plotted.

Displayed gel images were converted from raw data GEL to TIFF files in ImageJ. In Adobe Photoshop the contrast was enhanced using the levels setting and the images were cropped before import to Adobe Illustrator where figures were assembled.

## Supplementary information

Supplementary information

## Data Availability

Materials and strains are available upon request.
