# Supplementary material for: Either Rap1 or Cdc13 can protect telomeric single-stranded 3′ overhangs from degradation in vitro
Source: Sci Rep. 2019 Dec 16;9:19181. doi: 10.1038/s41598-019-55482-3 (PMC6915718; doi:10.1038/s41598-019-55482-3)
Supplement: Supplementary file 1 — Supplementary information [file 41598_2019_55482_MOESM1_ESM.pdf]

Supplementary material

Article title:

Either Rap1 or Cdc13 can protect telomeric single-stranded 3' overhangs from degradation *in vitro.*

Authors: Rikard Runnberg, Saishyam Narayanan†, Humberto Itriago† and Marita Cohn*

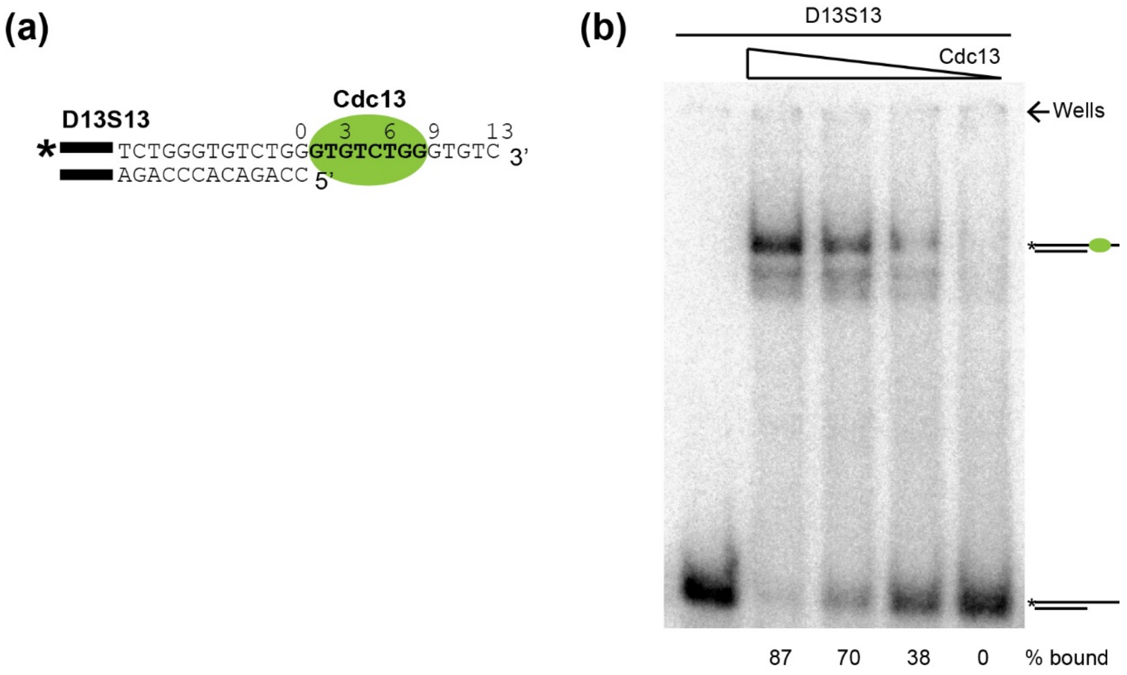

Figure S1. Binding of the Cdc13 protein to the D13S13 substrate. (**a**) Schematic showing D13S13 (i) with the expected interaction with Cdc13. Black bars indicate the 14 nt guide sequence for ensuring proper annealing, bold text indicates Cdc13 MBS, "*" indicates ^{32}P radioactive label. Numbers above the sequence denotes the distance in nt relative the ds-ss junction. (**b**) EMSA of D13S13 with a 2x dilution series of Cdc13 (second dilution was used for DEPAs presented in main manuscript). Schematics to the right of the gel indicates unbound substrate (lower band), Cdc13 bound substrate (up-shifted band) and the wells indicated by an arrow. Quantification of the percentage of bound probe is indicated below each lane.

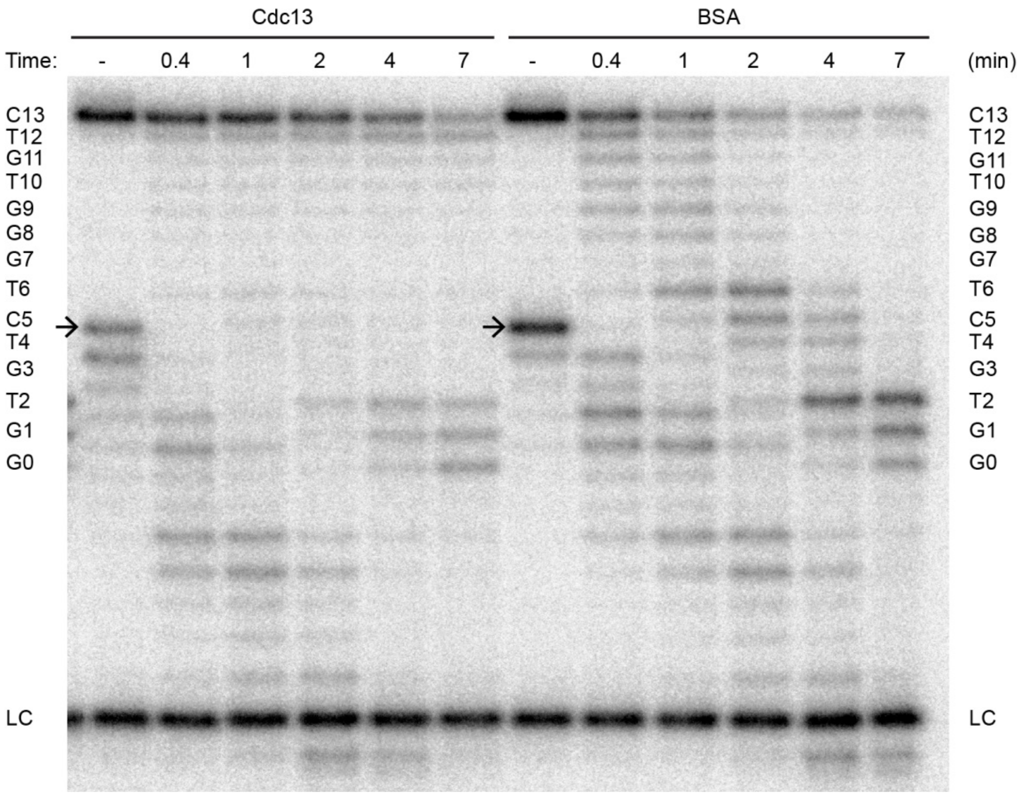

Figure S2. Protection of the 3’ overhang is dependent on Cdc13 DNA-binding. Sequencing gel showing 3’DEPA products of D13S13 incubated with 0.015 U/μl ExoT for 25 s, 1 min, 2 min, 4 min and 7 min, following pre-incubation with Cdc13 or BSA as indicated.”–“ indicates no enzyme added, “LC” loading control. The sequence of the 3' overhang is written beside the gel and numbered from the ds-ss junction (G0) to the 3’ end (C13). A 5’ end labelled non-telomeric oligonucleotide (5’-GGACTTAAAATGGCGTGGCAGAACTAACTCTT-3’) with no binding site for Cdc13 was added to the reaction in equal concentration to the substrate (marked with an arrow). Pre-incubation with Cdc13 slows down the rate of the digestion of the telomeric 3’ overhang substrate, while the digestion of the unbound non-telomeric oligonucleotide is quickly degraded when pre-incubated with both BSA and Cdc13, showing that the protection observed for D13S13 is dependent on the binding of Cdc13.

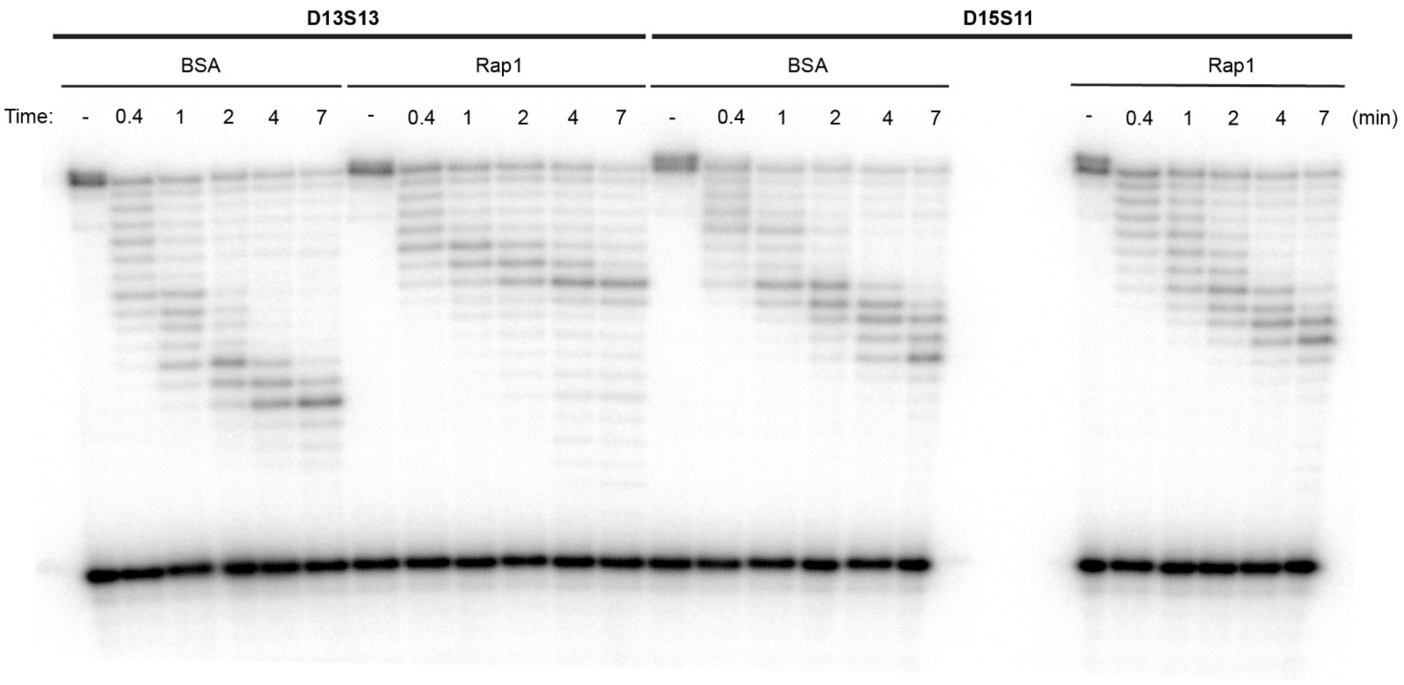

Figure S3. Uncropped gel of 3'DEPA's presented in Fig. 3 and Fig. 4 of the main manuscript. DEPA reactions with Rap1 using substrate D13S13 versus D15S11.

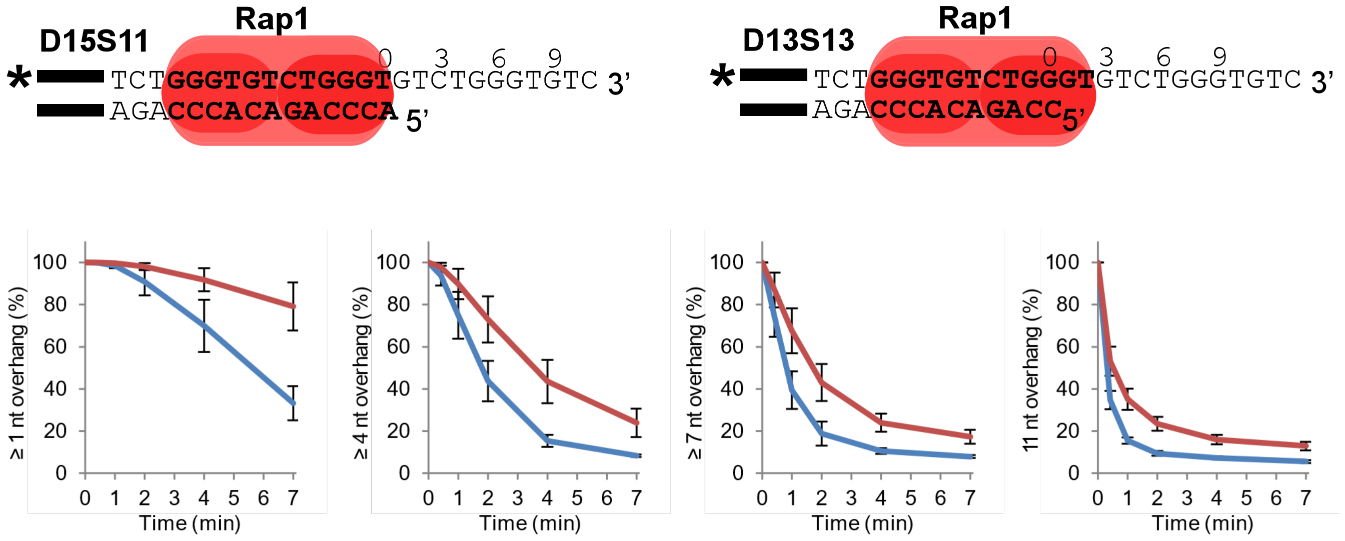

Figure S4. Quantification of the Rap1 protection at different positions of the 3' overhang (additional information to Fig. 3d in the main manuscript). Top schematic figure shows the binding of Rap1 to the D15S11 substrate. Graphs showing the proportion of D15S11 products at lengths of ≥1, ≥4, ≥7 and 11 nt, corresponding to positions at 1, 4, 7 and 11 nt of the 3' overhang, at each reaction time point for Rap1 (red line) and BSA (blue line). Error bars indicate SEM for n≥5 experiments.

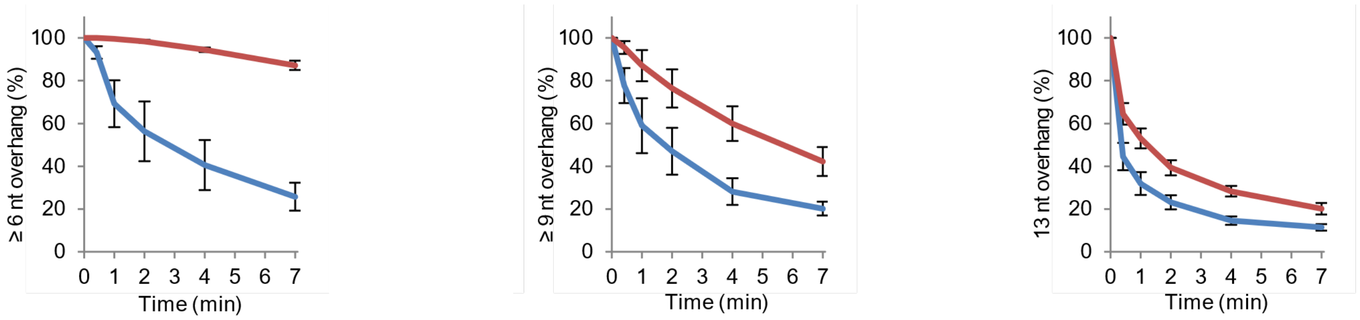

Figure S5. Quantification of the protection at different positions of the 3' overhang (additional information to Fig. 4d in the main manuscript). Top schematic figure shows the binding of Rap1 to the D13S13 substrate. Graphs showing the proportion of D13S13 products at lengths of ≥6, ≥9 and 13 nt, corresponding to positions at 6, 9 and 13 nt of the 3' overhang, at each reaction time point for Rap1 (red line) and BSA (blue line). Error bars indicate SEM for n≥6 experiments.

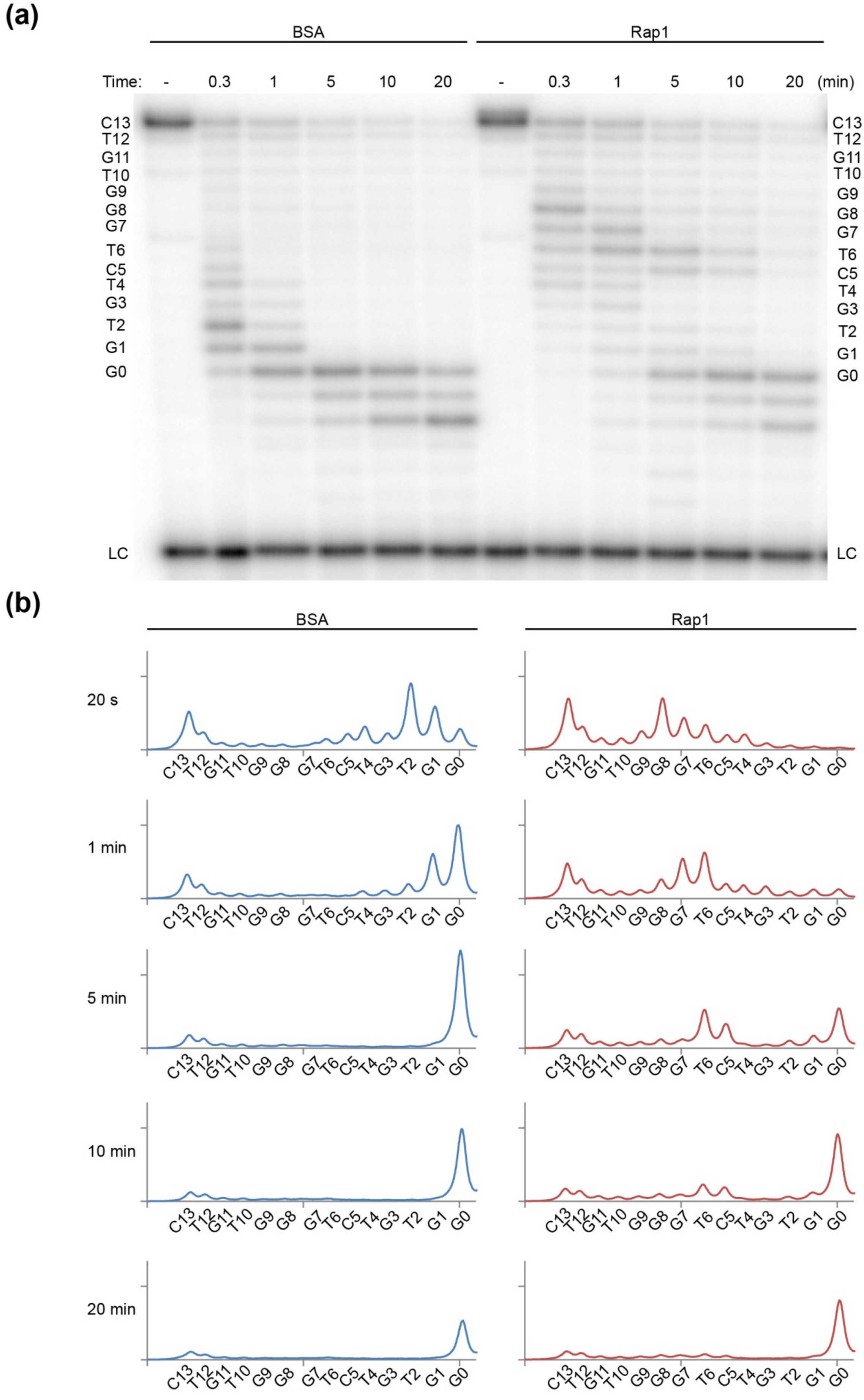
(a)
BSA
Rap1
Time: - 0.3 1 5 10 20 - 0.3 1 5 10 20 (min)
C13
T12
G11
T10
G9
G8
G7
T6
C5
T4
G3
T2
G1
G0
LC
(b)
BSA
Rap1
20 s
1 min
5 min
10 min
20 min
C13 T12 G11 T10 G9 G8 G7 T6 C5 T4 G3 T2 G1 G0

Figure S6. 3'DEPA shows protection of D13S13 by Rap1 also at higher amounts of ExoT and longer incubation times. (**a**) Sequencing gel showing 3'DEPA products of D13S13 incubated with 0.03 U/µl ExoT for 20 s, 1 min, 5 min, 10 min and 20 min, following pre-incubation with BSA or Rap1 as indicated."–" indicates no enzyme added, "LC" loading control. The overhang sequence beside the gel is numbered from the ds-ss junction (G0) to the 3' end (C13). (**b**) Lane profiles showing the intensities of each band in the lanes at the indicated reaction time points after pre-incubation with BSA (blue line) or Rap1 (red line). Nucleotide positions indicated as in (a).

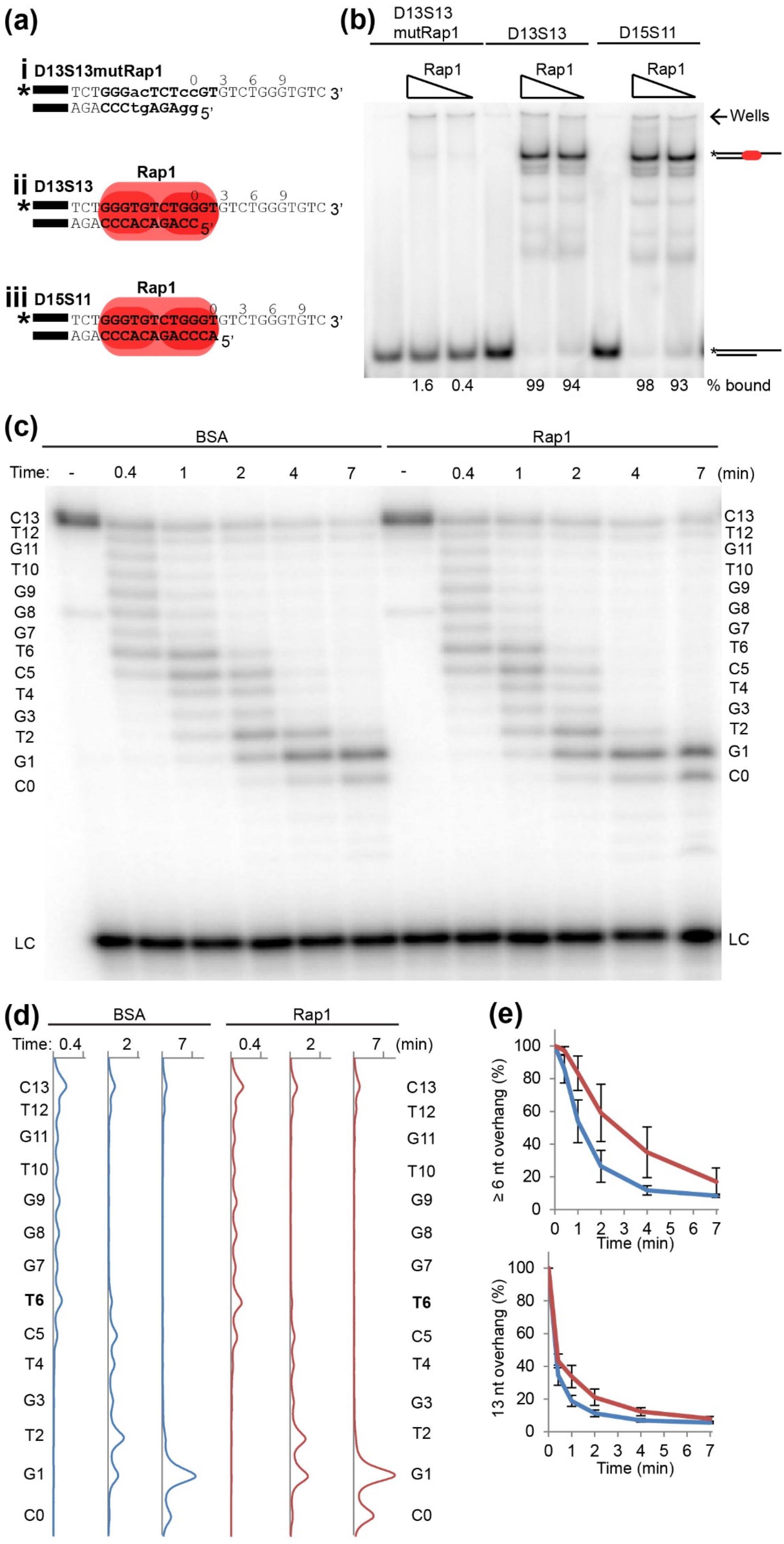

Figure S7. Unbound Rap1 does not stall ExoT. (**a**) Schematic showing D13S13mutRap1 (i), D13S13 (ii) and D15S11 (iii) with their expected interactions with Rap1, and lack thereof, respectively. D13S13mutRap1 (i) is identical to D13S13 (ii), except for four mutations (indicated by small letters) in the Rap1 MBS (bold letters). (**b**) EMSA of D13S13mutRap1, D13S13 and D15S11 with a 2x dilution series of Rap1 (same start dilution as used for DEPA). Schematics to the right of the gel indicates unbound substrate (lower band), Rap1 bound substrate (up-shifted band) and the wells indicated by an arrow. Quantification of the percentage of bound probe is indicated below each lane. (**c**) Sequencing gel showing 3'DEPA products of D13S13mutRap1 incubated with 0.015 U/µl ExoT for 25 s, 1 min, 2 min, 4 min and 7 min, following pre-incubation with BSA or Rap1 as indicated."–" indicates no enzyme added, "LC" loading control. The sequence of the overhang is numbered from the ds-ss junction (C0) to the 3' end (C13). (**d**) Lane profiles showing the intensities of each band in the lanes corresponding to the indicated time points for reactions with Rap1 (red line) or BSA control (blue line). The nucleotide position corresponding to each peak is indicated as in (c). (**e**) The proportion of overhangs at lengths of ≥6 and 13 nt was quantified at each reaction time for Rap1 (red line) and BSA (blue line), respectively. Error bars indicate SEM for n≥4 experiments.

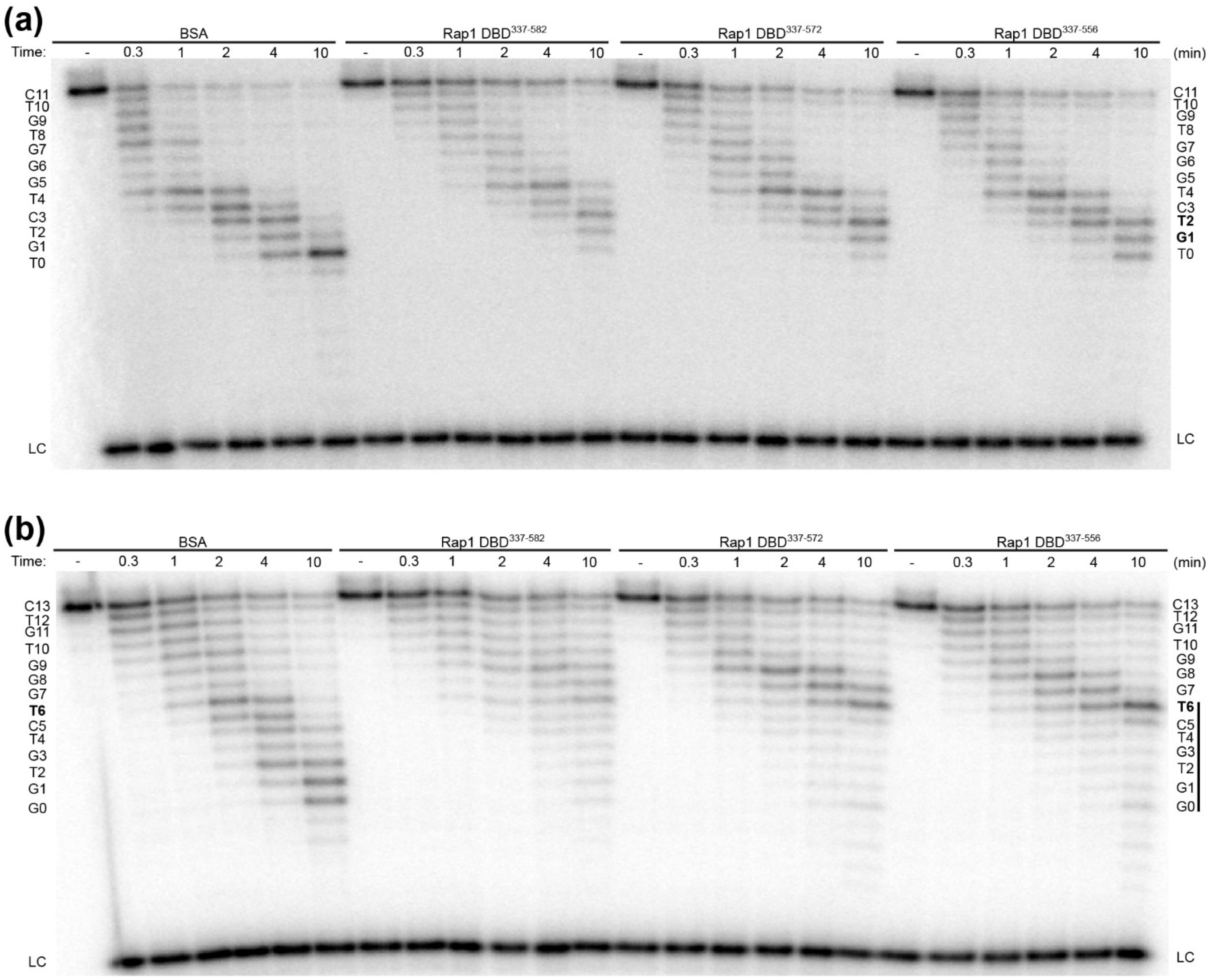

Figure S8. The Rap1 DBD is sufficient for protecting the 3' overhang against exonucleolytic degradation. Variants of the Rap1 DBD, containing the entire domain (DBD$^{337\text{-}582}$), lacking the latch region (DBD$^{337\text{-}572}$), or lacking both the wrapping loop and latch region (DBD$^{337\text{-}556}$), were pre-bound to D15S11 (**a**) or D13S13 (**b**), before incubation with 0.015 U/µl ExoT for 20 s, 1 min, 2 min, 3 min, 4 min or 10 min. "–" indicates no enzyme added. "LC" indicates loading control. The overhang sequence beside the gel is numbered from the ds-ss junction to the 3' end. Bold letters indicate bases protected by Rap1. The black bar next to the D13S13 sequence in (b) marks the area protected by Rap1. The gels show one representative experiment out of two independent experiments performed for each substrate.
